# Contrastive Multiscale Transformer for Image Dehazing

**DOI:** 10.3390/s24072041

**Published:** 2024-03-22

**Authors:** Jiawei Chen, Guanghui Zhao

**Affiliations:** School of Artificial Intelligence, Xidian University, Xi’an 710071, China; jiaweichen@stu.xidian.edu.cn

**Keywords:** image processing, image dehazing, deep learning, vision transformer, contrastive learning loss

## Abstract

Images obtained in an unfavorable environment may be affected by haze or fog, leading to fuzzy image details, low contrast, and loss of important information. Recently, significant progress has been achieved in the realm of image dehazing, largely due to the adoption of deep learning techniques. Owing to the lack of modules specifically designed to learn the unique characteristics of haze, existing deep neural network-based methods are impractical for processing images containing haze. In addition, most networks primarily focus on learning clear image information while disregarding potential features in hazy images. To address these limitations, we propose an innovative method called contrastive multiscale transformer for image dehazing (CMT-Net). This method uses the multiscale transformer to enable the network to learn global hazy features at multiple scales. Furthermore, we introduce feature combination attention and a haze-aware module to enhance the network’s ability to handle varying concentrations of haze by assigning more weight to regions containing haze. Finally, we design a multistage contrastive learning loss incorporating different positive and negative samples at various stages to guide the network’s learning process to restore real and non-hazy images. The experimental findings demonstrate that CMT-Net provides exceptional performance on established datasets and exhibits superior visual outcomes.

## 1. Introduction

Images acquired by cameras often exhibit severe degradation in hazy environments, including blurred scenes and color distortion [1]. These issues hinder the extraction of genuine image features by computer vision systems, consequently impacting subsequent analysis, understanding, recognition, and other processes [2,3]. Hence, the restoration of the original scene’s content and the enhancement of image readability through image dehazing hold significant practical value [4]. Atmospheric scattering physical models have been studied [5], in which the light received by the detection system originates from two sources: the light reflected by the target reaching the detection equipment after attenuation, and the atmospheric light resulting from light source scattering [6,7]. The final received image $I\left(x\right)$ can be described as follows:

(1) I(x)=J(x)t(x)+A(1−t(x))

where $J\left(x\right)$ denotes a clear image, A indicates the global atmospheric light, and *x* denotes the coordinates of each pixel in the image. The transmission map, $t\left(x\right)$, can be expressed as

(2) $$t(x)=e^{-\beta d(x)}$$

where $d\left(x\right)$ denotes the depth of the scene, and β represents the atmospheric scattering coefficient. Equations (1) and (2) can solve image restoration problems; therefore, by incorporating them into the model, non-hazy images can be recovered. Thus, the process of image dehazing can be construed as the restoration of a non-hazy image from its hazy counterpart. The key to dealing with dehazing is the estimation of $t\left(x\right)$ and A. The methods for estimating these parameters can be classified as prior- and neural network-based approaches.

Prior-based methods typically employ constraints to acquire atmospheric light and transmission maps [8,9,10,11,12]. These constraints are obtained using image processing methods or by utilizing the features contained in multiple hazy images. As deep learning continues to advance, convolutional neural networks (CNNs) can be used to learn A and $t\left(x\right)$ [13,14,15]. Cai et al. designed a network using a neural network-based method, and constructed a dehazing system using an end-to-end network. The network learns $t\left(x\right)$ and A to recover a dehazed image. This network has influenced future networks [16,17,18]. From the solution method for the atmospheric scattering model, we conclude that estimating $t\left(x\right)$ and A is a problem with an indefinite solution. The prior-based method is used in specific circumstances or obtained through experience, and the parameters obtained are inaccurate. Although reasonable values can be estimated using neural network-based methods, owing to the intricacy of real-world scenarios, the resulting dehazed image may have chromatic aberration. Moreover, in neural network-based methods, if one parameter is estimated incorrectly, the wrong parameter will continue to accumulate in the network, making it impossible to recover a non-hazy image. In recent years, CNN structures, instead of traditional atmospheric models, have been employed in end-to-end dehazing networks [19]. Liu et al. [20] introduced an attention-based multiscale network, GridDehazeNet, comprising preprocessing, backbone, and postprocessing components. Through these three steps, the features of the hazy images were learned to achieve reasonable dehazing. The ability of the CNN to learn features is excellent; however, in the inherent nature of the convolution operation, the CNN can only study potential and local hazy features. When a transformer is found to be helpful in computer vision [21], it is suitable for image restoration problems, such as image dehazing, because it needs to pay more attention to global information. Song et al. [22] used the transformer components to remove hazy images. After optimizing and improving the transformer, the network obtained better results in terms of haze removal. The apparent problem with the transformer is that it can only convey information in the same dimensions, which can significantly reduce the learning ability, making it less effective for image restoration tasks. Thus, the existing networks still have the following shortcomings:

1. Current CNN-based networks can learn only local features. A network that uses a transformer can only learn single-scale global information. This significantly limits the performance of these methods. Furthermore, the general network structure fails to extract the weight and feature information of the haze between different pixels and channels in hazy images with varying concentrations. It also fails to perceive and learn from noise in the form of haze. As the network depth increases, shallow image information may be degraded and lost.
2. In most networks, convolution typically uses a fixed-size kernel. When faced with irregular noise such as haze, information cannot be extracted well. In addition, current networks learn the features of non-hazy images, and one limitation of these methods is that they overlook the potential features present in hazy images. Therefore, a key challenge lies in the effective and comprehensive utilization of the information inherent in hazy images.

In this study, we use the proposed contrastive multiscale transformer method for image dehazing (CMT-Net) to address these shortcomings. We use a transformer to replace part of the structure of the existing CNN for dehazing tasks. By integrating the transformer into the traditional encoder–decoder structure, the network exhibits better generalization capabilities at different levels and scales. For the feature extraction of the image, we use an attention mechanism and deformable convolution to capture and express the distinctive characteristics of haze more comprehensively. For learning loss, we adopt the idea of contrastive learning, so that the network not only learns the characteristics of correct non-hazy images, but also avoid the characteristics of incorrect hazy images. Thus, the experimental results from the dehazing datasets effectively demonstrate the efficacy of CMT-Net for enhancing image dehazing performance. Our contributions are as follows:

- We propose a multiscale transformer with an encoder–decoder structure, which is connected using residual connections. This structure enables the network to fuse haze information from multiple scales and learn the global information of hazy images.
- We design a haze-aware module and add feature combination attention to the feature extraction, which enables the network to have a more vital transformation ability to fit the feature distribution of the haze.
- We design a novel multistage contrastive learning loss method. By employing different positive and negative sample pairs in the three stages, the network can effectively leverage information from both hazy and non-hazy images to achieve improved recovery consistency.

## 2. Related Work

As environmental pollution intensifies, image dehazing has become an important research topic. Two methods are currently used: prior- and neural network-based methods. In this section, we provide a concise overview of these methods.

### 2.1. Prior-Based Methods

Prior-based methods dehaze images by imposing constraints and restrictions on hazy images or prior information. Srinivasa et al. [5] established a haze model that explains the principles of haze and the definitions of various parameters. He et al. [8] observed multiple images and introduced dark channel prior (DCP) theory, in which each pixel has a small value in at least one of three channels in the non-sky region. The grayscale values of the top 0.1% of the pixels with the highest brightness in the image were considered atmospheric light; thus, hazy images can be restored by determining these two parameters. Choi et al. [23] designed a haze density estimator model that can be used for image dehazing. This method can directly predict the haze concentration from a single image without any additional information. Fattal et al. [24] combined color lines to predict and restore non-hazy images. Tan et al. [25] suggested that a clear image contrasts better than a hazy image. They determined that the value of atmospheric light is mainly related to the separation between the receiving device and target. Based on these two concepts, they established a cost equation for a Markov model and proposed a maximum-contrast dehazing method. Fattal et al. [26] proposed an independent component analysis color image dehazing algorithm. Zhou et al. [27] designed a dehazing algorithm that uses retinal cortex theory, and they convolved the image by designing a Gaussian filter to obtain atmospheric light. Zhang et al. [28] employed filtering with a high dehazing speed. Wang et al. [29] designed a multiscale retinal cortex and color restoration algorithm and considered the scene radiance of hazy images, for which the restored images have better detail. Ma et al. [30] proposed an image denoising method to remove real noisy images by using an unscented Kalman filter to simulate and estimate the noise. Since the distribution of haze is more difficult to fit than noise, it cannot be completely used for image dehazing. Prior-based methods have shown some success; however, they tend to rely heavily on specific environmental conditions. Thus, these methods do not effectively address complex situations.

### 2.2. Neural Network-Based Methods

Owing to the extensive integration of deep learning, methods based on neural networks have been widely utilized for image dehazing tasks. In the early stages, these methods utilized pairs of images to train the network and estimate two parameters within the atmospheric scattering model. Li et al. [16] integrated $t\left(x\right)$ and A into a single parameter, and the parameters were trained using a network to remove haze. Ren et al. [31] learned the $t\left(x\right)$ relation between non-hazy and hazy images and estimated another parameter in the DCP for dehazing. Zhang et al. [15] constructed a pyramidal network, which united $t\left(x\right)$, A, and non-hazy images to learn together. Numerous end-to-end networks have been proposed to address the insufficiency of atmospheric scattering models and the complexity of real scenes. Chen et al. [32] designed a network that utilizes global contextual information and progressive connection mechanisms to effectively remove haze from images and improve image quality. For a single network with poor performance, Qin et al. [33] enhanced image haze removal by utilizing feature fusion and attention mechanisms, thereby improving the overall image quality. Tu et al. [34] used a multi-scale transmission refinement mechanism and joint atmospheric light estimation to build a network. Liu et al. [35] developed a classical gradient descent method based on a built-in CNN to solve a variational model that can recover non-hazy images. Shao et al. [36] designed a dehazing network, obtaining better results by training the image translation and image-domain dehazing modules. Zhang et al. [37] used hazy concentration information for dehazing, and the atmospheric scattering model did not constrain their network. Zhang et al. [38] added semantic priors and adaptive feature fusion to a dehazing network to obtain better results. Dong et al. [39] designed a multiscale method using a U-shaped network. Chen et al. [40] designed a synthetic structure that is applicable to most dehazing models. In the dehazing task, because of the scarcity of hazy images, a generative confrontation network can play a significant role [41]. Song et al. [22] employed advanced neural network architectures and image enhancement techniques to remove haze and enhance image clarity. C2PNet utilizes a cycle-consistent adversarial network framework to remove haze from images and restore clear details, thereby improving visual quality [42]. Zhao et al. [43] introduced a two-stage weakly supervised network that utilizes the effective perceptual fusion strategy, resulting in clearer output images. Golts et al. [44] constructed an unsupervised image dehazing network using hazy images for training; the method adjusts the parameters using DCP.

Because transformers can capture long-range dependencies better than CNNs, transformers have also gradually found use in vision applications. Wang et al. [45] proposed a U-shaped mesh structure with superior dehazing. Liu et al. [46] used a Swin transformer for image restoration, which reduces the computational cost. Song et al. [22] introduced a pyramidal architecture transformer and downsampled to reduce the computing time. In contrast to the above methods, our network not only combines a CNN and transformer in a novel and reasonable manner, but also uses an attention mechanism to enhance the network’s performance. The strengths and weaknesses of each type of method are summarized in Table 1.

## 3. Proposed Method

In this section, we outline the flow and structure of CMT-Net. We explain the composition of the multiscale transformer, which serves as a fundamental building block for our network. Subsequently, we introduce the feature extraction component and explain the principle and function of each module. Finally, we develop a novel loss function specifically designed for network training.

### 3.1. Overview

**Figure 1:**
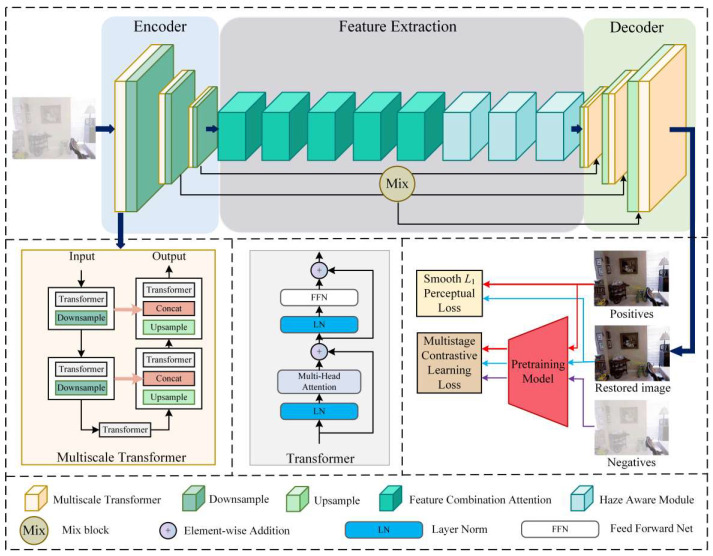
Illustration of proposed contrastive multiscale transformer for image dehazing (CMT-Net).

Image dehazing has always been a significant branch within computer vision because of its practical significance. To address current shortcomings, we propose a new network called CMT-Net, which is depicted in Figure 1. Overall, we add a multiscale transformer between each downsampling and upsampling. This structure can better learn the partial relationship and global relationship of the hazy information. Furthermore, we use feature combination attention and a haze-aware module in the feature extraction portion. These modules can enhance the adaptability of the network and provide deep dehazing. By setting a reasonable number of modules and order relationships, our network requires less memory storage. We also use a mixed block to avoid the loss of shallow information during network transmission. Finally, we introduce a novel multistage contrastive learning loss, which effectively obtains information from hazy images, thereby generating more natural-looking non-hazy images.

### 3.2. Multiscale Transformer

First, we briefly review Vision Transformer (ViT), which converts images into one-dimensional sequence representations, allowing the transformer to be used in image processing tasks [47]. In ViT, the transformer encoder consists of multi-head self-attention (MSA) layers and feedforward network (FFN) blocks, uses a layer norm (LN) before each block, and uses a residual connection after each block. The flow diagram is shown in Figure 1. The formula is summarized as follows:

(3) $$\hat{x}=x+MSA(LN(x))$$

(4) $$y=\hat{x}+FFN(LN(\hat{x}))$$

where x represents the feature map obtained after the patch embedding block, and $\hat{x}$ represents the intermediate feature map. The input feature map passes through an LN, then undergoes MSA, and is summed with itself to yield the intermediate output $\hat{x}$; it then passes through LN and FFN, and is summed again to obtain output y.

ViT has displayed very good performance in vision tasks. However, ViT can only learn the relationship between the local and global at one scale, and cannot transfer it to the deep layer. Therefore, we designed the multiscale transformer to compensate for these shortcomings. Our multiscale transformer adopts an encoder–decoder structure, as depicted in the lower-left part of Figure 1. In multiscale transformers, inspired by the Uformer, we employ a two-layer downsampling structure as our encoder and a two-layer upsampling structure as our decoder. Additionally, to facilitate effective information transmission across scales, we employ a residual connection to facilitate the aggregation of additional information. The utilization of a multiscale architecture ensures that the network can learn global image information, particularly by ensuring that the final non-hazy images are globally natural and realistic. First, for hazy images, we divide the images into small patches and position-encode them. The structure of haze in the image is understood through MSA, and then the network’s ability to represent haze is improved through nonlinear transformation and integration. By inputting and outputting the multiscale encoder and decoder, coarse and fine information in the image can be learned more accurately. Multiscale transformers have two advantages: they allow information transmission at different scales, and they facilitate better learning of haze characteristics and the extraction of global robust features. Below, we present the standardized architecture of the network and the specific details of the multiscale transformer in the form of Table 2.

### 3.3. Feature Extraction

In feature extraction, we utilize feature combination attention, which comprises five sets of channel and pixel attention. Based on these, three haze-aware modules were designed. Considering the deep structure of the network, we also added a mix block to ensure the effective transmission of shallow image information to deeper layers.

#### 3.3.1. Feature Combination Attention

**Figure 2:**
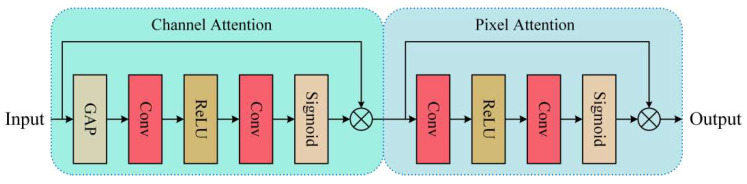
Feature combination attention process structure using a two-part attention process.

To address the challenge of fully extracting specific information from complex environments, we introduce feature combination attention. Through the analysis of haze features, we observe that haze exhibits different characteristics across channel and pixel positions. Therefore, inspired by the attention mechanism [48], we adopt channel and pixel attention to remove haze in a targeted manner. A structural flowchart is shown in Figure 2. Channel attention employs global average pooling to transform the feature maps into channel descriptors. To compute the weights of each channel, the features first undergo two convolutional layers and then pass through sigmoid and rectified linear unit (ReLU) activation functions. The input is multiplied element-wise by the channel weights, ensuring a strict transformation of the feature maps into compact channel descriptors. Similarly to channel attention, we pass two convolutional layers with ReLU and sigmoid activation functions and multiply them by the input to obtain the final output. Channel attention highlights channels that carry important information, whereas pixel attention accentuates features in regions with high haze concentrations. The process of pixel attention is similar to that of channel attention, but focuses on the weight information between pixels. This attention mechanism enables CMT-Net to concentrate more on densely hazy and high-frequency regions. By combining the two attention modules, significantly enhanced features are obtained.

#### 3.3.2. Haze-Aware Module

**Figure 3:**
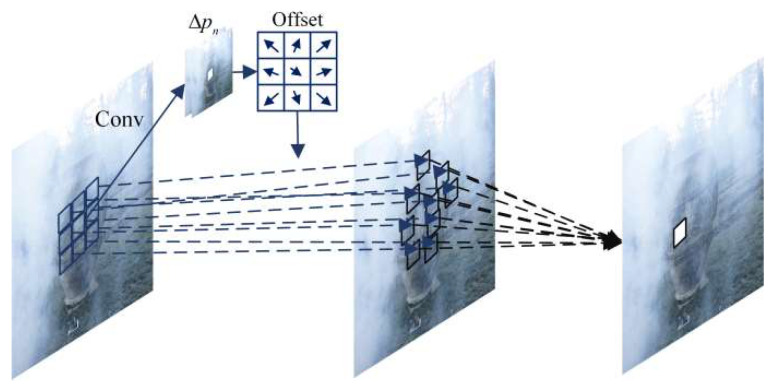
Illustration of the haze-aware module process.

In a dehazing network using a CNN, the features of hazy images are generally extracted through convolution operations. The geometric modeling ability of ordinary convolutional neural networks is limited by convolution, that is, using fixed deformations to deal with irregular and unknown deformations. Therefore, we require a module that can adaptively sense and accurately simulate the size of haze. Therefore, a haze-aware module was designed. The module can adaptively focus on the area of interest, which can lead to a real dehazing effect; the module deployed in the deep layer has a better effect than that deployed in the shallow layer and can fit the deep information of the haze. Next, we describe the haze-aware module in conjunction with Figure 3. We consider a 3×3 convolution as an example. If the center of the convolution is specified as (0,0), then the other positions can be expressed as {(−1, −1), (−1,0) … (0,1), (1,1)}, which can be expressed as

(5) $$y(p_{0})=\sum_{p_{n}\in R}w(p_{n})\cdot x(p_{0}+p_{n})$$

where $\sum_{p_{n}\in R}w(p_{n})$ denotes the weighted sum operation, $p_{0}$ represents the center of the convolution kernel, and $p_{n}$ denotes the offset of the remaining eight points relative to the center point, representing the convolution kernel operation. The haze-aware module we employ introduces an additional offset based on traditional convolution, which is expressed as follows:

(6) $$y_{HAM}(p_{0})=\sum_{p_{n}\in R}w(p_{n})\cdot x(p_{0}+p_{n}+\Delta p_{n})$$

In this formula, an additional offset is added to $p_{n}$, allowing the eight points to spread. In hazy images, this module serves as a replacement for conventional modules in the CNN and learns the offset through backpropagation. Our haze-aware module can extract the distribution and characteristics of haze, which cannot be achieved using traditional convolution.

#### 3.3.3. Mixed Block

**Figure 4:**
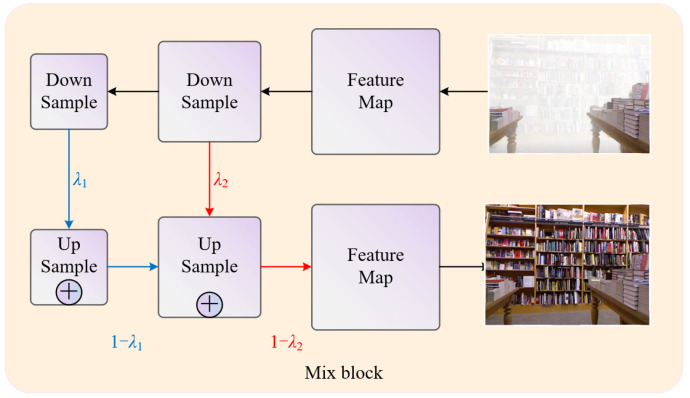
Illustration of the mixed block process.

While the network can learn detailed information in shallow layers [49], some information may be lost as the network depth increases. Although our network can learn features at different levels, there is a risk that shallow information may not be effectively transferred to the deep layers. Therefore, we introduce a mixed block to address this issue by mixing feature information from upsampling and downsampling, as shown in Figure 4. To ensure the optimal integration of features, we assign different weights to different upsampling and downsampling layers, surpassing the performance achieved using constant weights. This can be expressed as follows:

(7) $$F_{2}(Us)=\rho_{1}F_{1}(Ds)+(1-\rho_{1})F_{1}(Us)$$

(8) $$F_{out}=\rho_{2}F_{2}(Ds)+(1-\rho_{2})F_{2}(Us)$$

where $F_{i}$ represents information from different layers, Ds represents downsampling, Us represents upsampling, $F_{out}$ denotes the final output, and $\rho_{1}$ and $\rho_{2}$ are learned parameters. By mixing features of different scales, the final output enhances the texture details of the non-hazy image.

### 3.4. Multistage Contrastive Learning Loss

**Figure 5:**
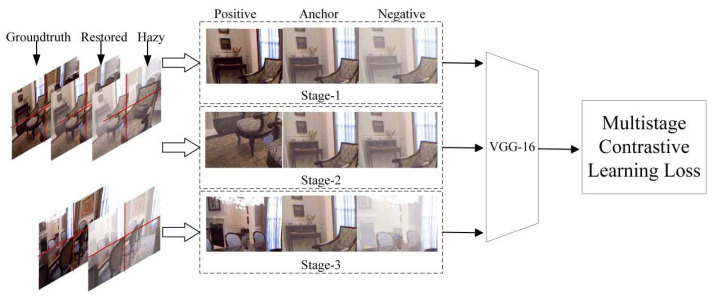
Illustration of the multistage contrastive learning loss process.

Recently, contrastive learning has gained widespread popularity in self-supervised learning. That is, for a given anchor point, the concept of contrastive learning involves pulling an anchor point closer to a positive point and simultaneously pushing it away from a negative point; therefore, the selection and number of positive and negative points are quite important [50]. Inspired by this idea [51], we used the contrastive learning method in the loss function to dehaze an image by defining different positive and negative samples in multiple stages. We cropped the restored image to one-quarter the size and selected a piece as the anchor; this is shown in Figure 5.

- The first stage uses a piece of the hazy image and a piece of the ground truth image corresponding to this image as positive and negative samples, respectively, to enable the network to better dehaze.
- In the second stage, the positive sample selects another piece of the restored image, and the negative sample selects a piece of the image after downsampling. This selection aims to ensure the consistency of image restoration and avoid haze in different periods.
- In the third stage, we select the real non-hazy image and the hazy image of another image from the dataset as the positive and negative samples, respectively. This means that we want the network to learn more about haze characteristics without paying attention to the image content.

The multistage contrastive learning loss can be formulated as

(9) $$L_{c}=-\sum_{i=1}^{N_{i}}\log[\frac{\sum_{j=1}^{N_{j}}\exp(r_{i}\cdot r_{j}/\tau )}{\sum_{j=1}^{N_{j}}\exp(r_{i}\cdot r_{j}/\tau )+\sum_{k=1}^{N_{k}}\exp(r_{i}\cdot r_{k}/\tau )}]$$

where $N_{i}$ represents the number of anchors, $N_{j}$ represents the number of positive samples, and $N_{k}$ represents the number of negative samples. The characteristics of the anchor, positive samples, and negative samples are denoted as $r_{i}$, $r_{j}$, and $r_{k}$, respectively. The features are extracted from the ninth layer of the pre-trained network model Visual Geometry Group network (VGG16) [52]; τ is the temperature parameter, which means that it can adjust the degree of attention of difficult samples of the model. The smaller τ is, the more the model focuses on separating those negative samples that are most like this sample.

The loss function of our network also uses $L_{smoothL1}$ and $L_{perceptual}$. We add a smooth $L_{1}$ loss, which has a more stable gradient than $L_{1}$ loss near zero, which makes the network more stable. The expression is as follows:

(10) $$L_{smoothL1}=\left\{\begin{array}{ll} 0.5E^{2} & if\left|E\right|<1\\ \left|E\right|-0.5 & otherwise, \end{array}\right.$$

where E=I−G, I is the dehazed image, and G is the real non-hazy image. We also use perceptual loss to improve the reconstruction of the restored image [53]. We use the features extracted from the fifth, seventh, and eleventh layers in VGG16 to calculate the perception. The total loss function is expressed as

(11) $$L=\lambda_{1}L_{smoothL1}+\lambda_{2}L_{perceptual}+\lambda_{3}L_{c}$$

where $\lambda_{1},\lambda_{2},\lambda_{3}$ are parameters that balance the importance of different losses, which can make the loss function more suitable for the dehazing task.

## 4. Experiments

This section introduces the hazy datasets on which the networks were trained. We illustrate other methods for comparing the experimental results and introduce evaluation metrics. The efficacy of the proposed network is evaluated through metric comparisons. We also analyzed the experimental results to demonstrate the effectiveness of CMT-Net and each module through ablation experiments. In addition, we applied the proposed multiscale transformer and multistage contrastive learning loss to other networks to demonstrate its universality.

### 4.1. Datasets

**Figure 6:**
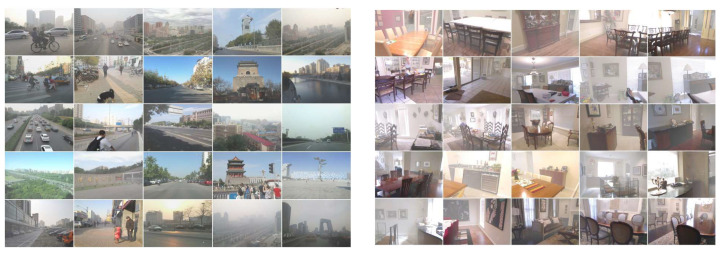
Example images from the five sets in OTS and SOTS datasets.

**Figure 7:**
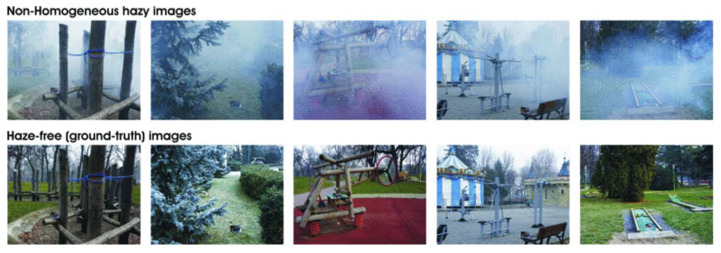
Five pairs in the example images in NH-HAZE.

The datasets we use are existing recognized image dehazing datasets. We introduce the datasets used by CMT-net in Realistic Single Image Dehazing (RESIDE) [54]. We used two of the datasets, namely, the outdoor training set (OTS) and the synthetic objective testing set (SOTS). OTS was employed for training under outdoor conditions, whereas SOTS functioned as a unified testing set for both indoor and outdoor scenarios. We utilized the NH-HAZE dataset [55], consisting of 45 pairs of real-world images with varying degrees of haze. Unlike previous datasets, this collection contains both thin and dense hazy regions within each image. We used 8500 images from the OTS to train our network and 50 images for testing. Within the SOTS dataset, 480 images were used for training and 20 for testing. In the NH-HAZE dataset, 40 sets of images were used for training and 5 for testing. Examples of these two datasets will be shown in Figure 6 and Figure 7. In addition, we also conducted experiments using currently popular real-world hazy images to demonstrate that the images restored by our network are more realistic and natural. Network performance was demonstrated by averaging the metrics obtained from the tested images.

### 4.2. Comparison Methods and Metrics

To substantiate the effectiveness of our proposed network, we chose widely used methods for comparison, namely, the prior-based method DCP (TPAMI’10) [8], and the neural network-based methods DCPDN (CVPR’18) [15], RefineDNet (TIP’2021) [43], AECRNet (CVPR’21) [49], Maxim (CVPR’2022) [34], DehazeFormer (TIP’2022) [22], and C2PNet (CVPR’2023) [42]. We used two popular evaluation metrics as references: the Dehazing Quality Index (DHQI) [56] and the “completely blind” Natural Image Quality Evaluator (NIQE) [57]. The DHQI measures the quality of an image from three sets of features. In the haze-removing stage, DCP was employed to estimate the haze density, and in the structure-preserving stage, contrast was used to evaluate the image quality. Finally, the mean and covariance were used in the over-enhancement stage to determine whether the image contained artifacts. The NIQE aims to build a feature set for assessing the image quality and then fits these features to a multivariate Gaussian model (MVG). The distance can be expressed as

(12) $$D(V_{1},V_{2},\sigma_{1},\sigma_{2})=\sqrt{\left({\left(V_{1}-V_{2}\right)}^{T}{\left(\frac{\sigma_{1}+\sigma_{2}}{2}\right)}^{-1}(V_{1}-V_{2})\right)}$$

where $V_{1}$, $V_{2}$, $\sigma_{1},$ and $\sigma_{2}$, respectively, represent the mean vector and covariance matrix of the restored image in the MVG model, and the pre-trained image in the MVG. In addition, we utilized the peak signal-to-noise ratio (PSNR) and structural similarity (SSIM). The formulas used are as follows:

(13) $$\mathrm{MSE}=\frac{1}{M\cdot N}\sum_{x=0}^{M-1}\sum_{y=0}^{N-1}\left[I(x,y)-G(x,y)\right]$$

(14) $$\mathrm{PSNR}(I,G)=10\times \log_{10}(\frac{{\mathrm{MaxValue}}^{2}}{\mathrm{MSE}})$$

(15) $$\mathrm{SSIM}(I,G)=\frac{\left(2\mu_{I}\mu_{G}+c_{1})(\sigma_{\mathrm{IG}}+c_{2}\right)}{\left(\mu_{I}^{2}+\mu_{G}^{2}+c_{1})(\sigma_{I}^{2}+\sigma_{G}^{2}+c_{2}\right)}$$

for an image with a size of M·N, where I is a hazy image, G is a real non-hazy image, and x and y represent the horizontal and vertical coordinates. MaxValue is the maximum value of the image pixels. In the calculation formula of SSIM, μ is the standard deviation, σ is the covariance, and $c_{1}$ and $c_{2}$ are non-zero constants. Furthermore, to enhance the assessment of the computational complexity and parameter quantity of CMT-Net, we have included metrics such as parameters, multiply–accumulate operations (MACs) and running time. These metrics serve to elucidate the computational complexity of the algorithm.

### 4.3. Implementation Details

We conducted the experiments using PyTorch 1.9.0 on an Nvidia 1660 SUPER GPU from Santa Clara, United States. The model employed the Adam optimizer with the following parameter settings: $\beta_{1}$ = 0.9, $\beta_{2}$ = 0.999, and an initial learning rate of 0.001. The parameters in the loss function were set to $\lambda_{1}$ = 1, $\lambda_{2}$ = 0.5, and $\lambda_{3}$ = 0.1.

### 4.4. Image Results Analysis

#### 4.4.1. Qualitative Analysis

**Figure 8:**
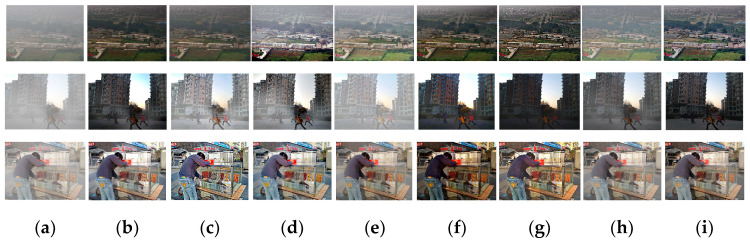
Dehazing results on different datasets. (**a**) Hazy image, (**b**) dark channel prior (DCP), (**c**) DCPDN, (**d**) RefineDNet, (**e**) AECRNet, (**f**) Maxim, (**g**) DehazeFormer, (**h**) C2PNet and (**i**) CMT-Net.

**Figure 9:**
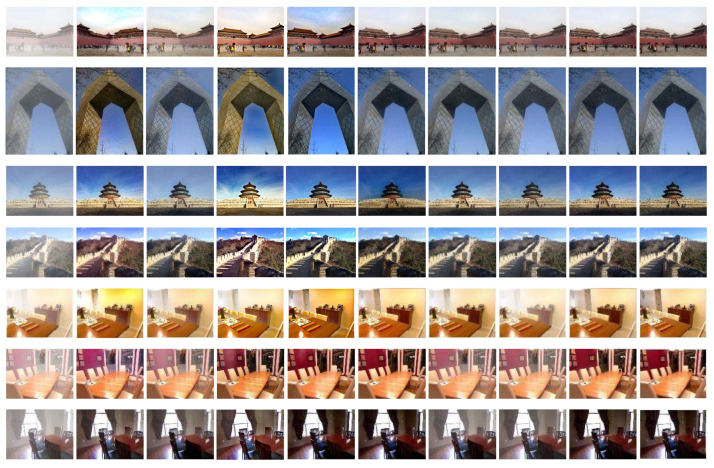

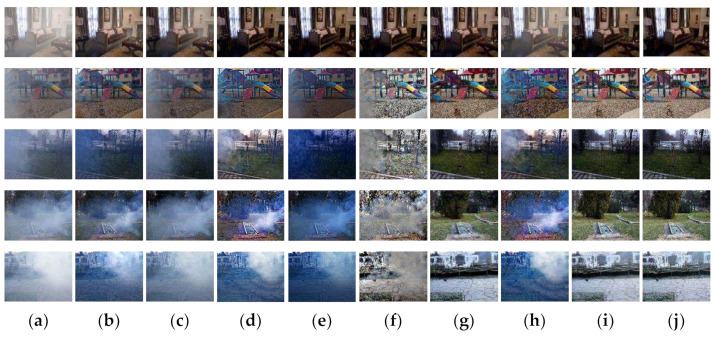
Dehazing results on different datasets. (**a**) Hazy image, (**b**) dark channel prior (DCP), (**c**) DCPDN, (**d**) RefineDNet, (**e**) AECRNet, (**f**) Maxim, (**g**) DehazeFormer, (**h**) C2PNet, (**i**) CMT-Net and (**j**) ground truth image.

First, the three image sets in Figure 8 consist of real hazy images. Upon visual inspection and comparison with the reference algorithm, it is evident that the images processed by CMT-Net exhibit complete removal of haze residue. The dehazing performed by CMT-Net is notably more thorough within the depth of field region, preserving a greater amount of scene detail and enhancing overall realism and natural color rendition. Then, we conducted a qualitative analysis of the performance of the algorithm using the following two datasets. The first eight sets of images were synthetic datasets, and two datasets, OTS and SOTS, were selected for testing. The resulting images are illustrated in Figure 9. The first is a prior-based method; because of model limitations, DCP cannot normally deal with sky haze. This is generally performed using synthetic indoor datasets. Following the DCPDN, we found that the haze removal results were incomplete. In RefineDNet, the color of the image after dehazing is distorted. The dehazing performance in AECRNet is poor, and the contrast of the image is excessively enhanced. The results after the maximum treatment were better, and no haze residue can be seen. DehazeFormer also achieved satisfactory results; however, residual haze remained in these images. C2PNet achieved good results, but the restored image had an extremely light haze layer; therefore, the effect was not good. Finally, in our CMT-Net, we carefully compared the processed results and believe that our method achieves good results with no residual haze when dealing with haze in the sky region and the depth of field. Moreover, our restored image exhibited no color discrepancies and closely resembled a clear image. We then evaluated CMT-Net on a real-world dataset consisting of two types of images, moderate haze and thick haze, to assess the universality of our proposed network. The DCP shows evident dehazing effects in thin haze regions, but struggled with dense haze areas. Similarly, DCPDN exhibits better dehazing results in thin haze regions, but leaves residual haze in dense haze areas. RefineDNet and AECRNet cannot handle situations with different haze concentrations well, and dense haze still exists in the image after dehazing. However, it may suffer from low contrast in the restored images. Maxim and C2PNet can remove part of the haze; however, there is a significant color difference compared with the real image. DehazeFormer performed well in this dataset by removing dense haze, but lost some texture details. In contrast, our network successfully removed haze of varying concentrations and restored the color and texture details of the original image.

#### 4.4.2. Quantitative Analysis

We conducted a quantitative analysis to demonstrate the superiority of CMT-Net by employing the metrics DHQI, NIQE, PSNR, and SSIM. The results are presented in Table 3. The table shows that apart from a suboptimal value in the DHQI, our network outperforms the others in all indicators. Particularly for the OTS dataset, we achieved remarkable results with a PSNR of 30.34 and an SSIM of 0.958, surpassing other methods by a significant margin. Moving on to the real-world dataset presented in Table 4, the haze concentration varied by location. Except for the DehazeFormer method, the performance of the other methods was relatively ordinary and significantly inferior to that of the synthetic dataset. However, even when compared to DehazeFormer, our network still achieved superior results across all indicators. These metrics further validate that CMT-Net produces more realistic and real non-hazy images that closely resemble the true images. While CMT-Net demonstrated favorable results in dehazing evaluation metrics and comparison with haze-free images, it did not yield the most optimal outcomes in terms of parameter size and MACs. This can be attributed to the presence of multiple layers of transformer within the network. Moreover, although our network may not exhibit the fastest running time, it remains proficient in addressing dehazing tasks that do not necessitate real-time performance.

### 4.5. Ablation Study

**Figure 10:**
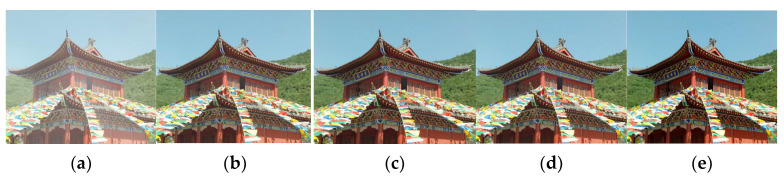
Ablation experiment dehazing results. (**a**) Hazy image, (**b**) Base (**c**) Base + HAM +MB, (**d**) Base + HAM + MB + MT, (**e**) Base + HAM + MB + MT + MCLL.

To assess the usefulness and justification of each module in CMT-Net, we performed ablation experiments. These modules include a multiscale transformer (MT), haze-aware module (HAM), mixed block (MB), and multistage contrastive learning loss (MCLL). We begin by considering the following base modules: upsampling, downsampling, and five feature combination attention modules. We then tested the network by adding each module individually and compared the results of different indicators to confirm its practicality. The experimental configurations were as follows: (1) Base: upsampling, downsampling, and five feature combination attention modules; (2) Base + HAM + MB: addition of three haze-aware modules and a mixed block; (3) Base + HAM + MB + MT: incorporating the multiscale transformer into the sampling part; and (4) Base + HAM + MB + MT + MCLL: multistage contrastive learning loss in the network. The results of each model are shown in Figure 10.

Table 5 shows that the base framework achieves a PSNR of 24.75 and an SSIM of 0.885. This demonstrates the effectiveness of our feature combination, which outperformed other network methods on certain datasets. The addition of haze-aware modules and mixed blocks led to noticeable improvements, indicating that the network became more adaptive to haze characteristics and could effectively transfer shallow edge details to deeper layers. Furthermore, the addition of the multiscale transformer enabled the network to study global haze information, whereas the inclusion of multistage contrastive learning loss resulted in the highest PSNR and SSIM values of 30.42 and 0.949. This indicates that the restored image was closer to the real image. Each module used in our network serves its own purpose in the dehazing process, and its integration enhances the overall dehazing performance. Among these, the multiscale transformer and haze-aware module make significant contributions, whereas the multiscale transformer and multistage contrastive learning loss improve the indicator performance and can be fused with other networks to enhance their effectiveness. Subsequently, we embedded these two modules into other networks for testing.

The ablation experiments were performed using a multiscale transformer. We integrated this module into the multilayer structure of a dehazing network and validated its effectiveness by comprehensively testing the key performance indicators. From Table 6, we can observe that by adding it to the other networks, both the PSNR and SSIM indicators were improved. This is because the multiscale transformer structure can expand the overall information of more images and pass them deep into the network.

Through ablation experiments, it was proven that the designed multistage contrastive learning loss is practical and can better guide the dehazing process of the entire network. To demonstrate the practicality and convenience of the multistage contrastive learning loss function, we applied it to different networks. As shown in Table 7, both the PSNR and SSIM improved. After using this loss, the loss of image information can be reduced and the image details can be preserved. Furthermore, our loss function can not only be used in image dehazing, but can also be extended to some low visual tasks.

## 5. Conclusions

In this study, we proposed a novel image dehazing network called CMT-Net. We used the multiscale transformer module of the encoder–decoder structure to learn and fuse the multiscale features of haze. By adding feature combination attention and haze-aware modules, haze had more weight in feature mapping and improved the adaptability of the network. Finally, we designed a new multistage contrastive learning loss function that facilitates superior recovery of non-hazy images. The proposed loss function can also be extended to other networks. Numerous experiments demonstrated that the proposed CMT-Net achieves superior performance across various datasets. In a subsequent study, we will focus on developing and designing lighter network architectures and applying them to other image processing tasks.

## Figures and Tables

**Table 1:** The applicable situations, strengths and weaknesses of previous methods and CMT-Net.

Method	Strengths	Weaknesses
Prior-based method [8,23,24,26,27,28,29,30]	Wide applicability, fast calculation, and good visual effect after restoration.	Unable to remove haze in dense haze areas, resulting in color distortion.
Network based on physical models [15,16,31,34,39,42]	Only the parameters related to the physical model need to be learned, and the computational data are limited. After restoration, the haze in the image is basically removed.	For real and complex environments that do not satisfy prior conditions, it is impossible to remove haze, and if errors occur when calculating parameters in the physical model, the errors will accumulate, leading to the loss of information in the restored image.
Network based on CNN structure [32,33,34,35,36,37,38]	The CNN structure can learn features well, has a wide range of applicability, and can directly generate haze-free images end-to-end.	CNNs cannot capture the global information of images, and there may be overfitting issues. There is no targeted module for characterizing and learning the irregular noise of haze.
Network based on transformer [22,45,46]	Transformer can capture global information of images and has strong parallelism, which is more conducive to network training, and can adapt well to complex images.	Transformer requires more computing resources and can only learn global information at a single scale, making it impossible to transmit at different scales. In addition, the potential information of hazy images is ignored, and there is a lack of modules for learning and fitting haze.
CMT-Net (ours)	For hazy areas, haze can be completely removed, and the information from hazy images can be utilized to improve recovery consistency. The restored image is more realistic and natural, while preserving the original scene information and texture details.	The network computing cost is high, and the processing effect for hazy images at night is not good, and the network’s ability is limited by the number of datasets.

**Table 2:** Detailed architectural specifications for multiscale transformer and the overall network.

Block	Layer	Input	Output
Multiscale Transformer	Transformer_1	C × W × H	C × W × H
	Downsample_1	C × W × H	2C × W/2 × H/2
	Transformer_2	2C × W/2 × H/2	2C × W/2 × H/2
	Downsample_2	2C × W/2 × H/2	4C × W/4 × H/4
	Transformer_3	4C × W/4 × H/4	4C × W/4 × H/4
	Upsample_2	4C × W/4 × H/4	2C × W/2 × H/2
	Concat_2	2C × W/2 × H/2	2C × W/2 × H/2
	Transformer_2	2C × W/2 × H/2	2C × W/2 × H/2
	Upsample_1	2C × W/2 × H/2	C × W × H
	Concat_1	C × W × H	C × W × H
	Transformer_1	C × W × H	C × W × H
Encoder–Decoder	Multiscale Transformer_1	C × W × H	C × W × H
	Downsample_1	C × W × H	2C × W/2 × H/2
	Multiscale Transformer_2	2C × W/2 × H/2	2C × W/2 × H/2
	Downsample_2	2C × W/2 × H/2	4C × W/4 × H/4
	Multiscale Transformer_3	4C × W/4 × H/4	4C × W/4 × H/4
	Downsample_3	4C × W/4 × H/4	8C × W/8 × H/8
	Feature Extraction	8C × W/8 × H/8	8C × W/8 × H/8
	Upsample_3	8C × W/8 × H/8	4C × W/4 × H/4
	Multiscale Transformer_3	4C × W/4 × H/4	4C × W/4 × H/4
	Upsample_2	4C × W/4 × H/4	2C × W/2 × H/2
	Multiscale Transformer_2	2C × W/2 × H/2	2C × W/2 × H/2
	Upsample_1	2C × W/2 × H/2	C × W × H
	Multiscale Transformer_1	C × W × H	C × W × H

**Table 3:** Quantitative comparison of the Realistic Single Image Dehazing (RESIDE) dataset based on Dehazing Quality Index (DHQI), Natural Image Quality Evaluator (NIQE), peak signal-to-noise ratio (PSNR), structural similarity (SSIM), param, MACs and runtime.

Method	RESIDE		OTS		SOTS		Param (M)	MACs (G)	Run Time(s)
	DHQI ↑	NIQE ↓	PSNR ↑	SSIM ↑	PSNR ↑	SSIM ↑			
DCP	44.722	4.312	14.65	0.734	15.72	0.692	-	-	19.36
DCPDN	50.975	4.128	21.15	0.842	18.69	0.815	66.89	0.581	24.94
RefineDNet	51.725	3.842	26.45	0.907	18.75	0.835	65.80	26.24	0.77
AECRNet	49.453	3.712	17.54	0.842	19.43	0.829	2.61	28.08	0.87
Maxim	**66.167**	3.578	28.99	0.934	20.73	0.889	14.1	216	15.53
DehazeFormer	59.963	3.541	29.23	0.945	19.75	0.852	80.45	48.93	0.12
C2PNet	52.735	4.072	28.75	0.929	17.86	0.781	9.23	7.17	1.23
CMT-Net	63.452	**3.501**	**30.34**	**0.958**	**21.85**	**0.907**	39.45	34.16	1.56

The symbol ↑ means higher is better, ↓ means lower is better. **Bold** and red correspond to the best and second-best results, respectively.

**Table 4:** Quantitative comparison of the NH-HAZE dataset based on DHQI, NIQE, PSNR, SSIM and runtime.

Method	NH-HAZE		Thin Haze		Dense Haze		Run Time(s)
	DHQI ↑	NIQE ↓	PSNR ↑	SSIM ↑	PSNR ↑	SSIM ↑	
DCP	35.87	6.154	14.94	0.713	13.59	0.531	22.61
DCPDN	30.24	6.231	13.82	0.597	11.79	0.422	25.13
RefineDNet	33.15	6.638	11.58	0.624	14.75	0.529	0.77
AECRNet	36.79	5.759	16.29	0.741	13.96	0.546	0.943
Maxim	32.06	5.724	14.90	0.697	11.66	0.413	17.45
DehazeFormer	42.26	4.956	27.89	0.902	26.73	0.807	0.15
C2PNet	38.91	5.687	14.56	0.682	11.73	0.438	1.37
CMT-Net	**45.72**	**4.764**	**28.11**	**0.923**	**26.98**	**0.854**	1.43

The symbol ↑ means higher is better, ↓ means lower is better. **Bold** and red correspond to the best and second-best results, respectively.

**Table 5:** Ablation study on CMT-Net.

Model	PSNR	SSIM	Param (M)	MACs (G)
Base	24.75	0.885	0.956	21.54
Base + HAM +MB	26.63	0.902	2.611	22.21
Base + HAM + MB + MT	29.91	0.937	26.75	28.93
Base + HAM + MB + MT + MCLL	**30.42**	**0.949**	41.17	30.52

**Bold** corresponds to the best results.

**Table 6:** Results of using multiscale transformers in other networks.

Method	PSNR	SSIM
GCANet	25.25 (↑ 2.35)	0.852 (↑ 0.037)
C2PNet	16.64 (↑ 1.21)	0.713 (↑ 0.021)
DCPDN	18.72 (↑ 0.96)	0.831 (↑ 0.014)

The symbol ↑ represents a rising value.

**Table 7:** Results of using multistage contrastive learning loss in other networks.

Method	PSNR	SSIM
DCPDN	19.25 (↑ 0.35)	0.752 (↑ 0.018)
GridDehazeNet	16.64 (↑ 0.41)	0.713 (↑ 0.011)
FFA-Net	18.72 (↑ 0.66)	0.831 (↑ 0.024)

The symbol ↑ represents a rising value.

## Data Availability

The RESIDE dataset and NH-HAZE dataset can be found here: https://sites.google.com/view/reside-dehaze-datasets (accessed on 24 August 2023) and https://data.vision.ee.ethz.ch/cvl/ntire20//nh-haze/ (accessed on 24 August 2023).
